# A Novel Approach to Immediate Implants: The CastleWall Surgical Technique

**DOI:** 10.3390/dj10040062

**Published:** 2022-04-06

**Authors:** Cameron Castle

**Affiliations:** Private Practice, CPD, 76 Woongarra St., Bundaberg, QLD 4670, Australia; cameroncastle11@gmail.com; Tel.: +61-400087660

**Keywords:** socket-shield, partial extraction therapy, CastleWall, immediate implant

## Abstract

Objective: The purpose of this study was to investigate the volumetric stability around immediate implants, in which a 360-degree socket-shield was retained using the CastleWall Surgical Technique (CWST). Material and methods: This retrospective study examined the results of the CWST used for 25 consecutive patients, involving 31 immediate implants. Silicone impressions taken prior to extraction, and at a review appointment were converted to STL files and compared. The median follow-up time was 14.2 ± 5.5 months. Volumetric changes and gingival recession on both buccal and lingual sites were measured. Papillary height changes were also evaluated from available photographs taken before and after treatment. Patients in the study completed a Visual Analogue Scale (VAS) for evaluation of post-operative discomfort and overall satisfaction with this procedure. Results: All implants integrated successfully without complications. Mean loss of buccal and lingual tissue was 0.30 ± 0.32 mm and 0.17 ± 0.27 mm, respectively. Mean recession at the mid-buccal and mid-lingual gingival margin was 0.66 ± 0.64 mm and 0.87 ± 0.84 mm, respectively. Mean recession of the mesial and distal papilla was 0.26 ± 0.55 mm and 0.29 ± 0.52 mm, respectively. Patients reported 97.74 ± 5.60% satisfaction with this procedure using the Visual Analogue Scale (VAS), with minimal post-operative discomfort. Conclusions: The results of this study showed excellent soft tissue stability and aesthetics were achieved using the CWST, with minimal postoperative pain. The other main advantage of retaining a 360-degree socket-shield, is there is more available surface area to lock the implant to the shield to prevent shield migration over time.

## 1. Introduction

The complete extraction of a tooth root prior to implant placement launches a cascade of unintended and irreversible biological consequences. Soft tissue recessions and changes accompany the fast resorbing underlying bony scaffold [1,2]. Many proposed techniques such as bone substitute material grafting, guided surgery, platform switching and immediate implantation have failed to offset this invariable bone resorption following a complete extraction [3,4,5,6,7,8,9,10,11].

In 2010, Hürzeler et al. described a new method in an attempt to mitigate this bone resorption by retaining a buccal root segment or “Socket-Shield” at the time of implant placement [12]. There is currently no consensus in the literature on how far this shield should extend interproximally. Kan et al. in 2013 proposed a “Proximal Socket-Shield”, in an effort to preserve the proximal papilla adjacent to pre-existing implant restoration [13]. Cherel et al. described a similar technique [14]. A case report by S. Aslan showed significant palatal atrophy when a buccal shield was used [15]. 

With the current literature available, it may be intuitively deduced that tissue atrophy may occur where the shield does not extend. By increasing the circumference of the shield from a partial buccal shield to a continuous full 360-degree shield, soft tissue stability may be potentially improved in both the interproximal and palatal regions.

In 2014, Troiano et al. described a successful clinical study utilising a 360-degree shield, which Troiano termed the “Root-T-Belt” procedure [16]. The osteotomy was performed directly through the root and the implant placed [16]. Animal studies have also confirmed the biological success of using a 360-degree shield [17]. 

The difficulty in preparing the osteotomy through the root is ensuring the complete removal of the apex. Drill bits are circular and straight whereas a tooth root is often curved and elliptical in cross-section. Rotating drill bits also have a tendency to drift from a dense tooth root into less dense bone. This may be a problem in certain sites such as on the anterior maxilla where the bone can be very thin and easily perforated [18,19]. A different and novel method of preparing a 360-degree shield known as the “CastleWall Surgical Technique” (CWST) was developed to overcome the limitations of the method described by Troiano et al. [16].

Current literature also indicates that one of the biggest problems associated with using a socket-shield technique is “shield migration” [3,20]. Movement of the shield over time invariably leads to exposure of the shield which may necessitate complete removal [21]. Surgical removal of the shield may compromise both tissue stability and aesthetics negating any benefits afforded by using such a technique in the first place. The main advantage of preparing a larger diameter shield is the increased available surface area to “lock” the implant against the shield to prevent future shield movement and migration.

The aim of this study was to investigate soft tissue stability using the CWST and its effect on patient-based outcomes. 

## 2. Materials and Methods

This retrospective study covered a period of 24 months in a private practice, and included all patients that received immediate implants using the CWST. In this study a total of 31 immediate implants were placed consecutively in 25 patients, which included 11 males and 14 females. The median age of the patients was 64 ± 9.35 years at the time of surgery, and median follow-up time for this study was 14.2 ± 5.5 months after treatment.

Inclusion of patients for this retrospective study was determined by criteria as shown in Table 1. All patients involved in this study were provided with a range of treatment options, including the advantages and disadvantages of each option. 

### 2.1. Surgical Technique

**Figure 1:**
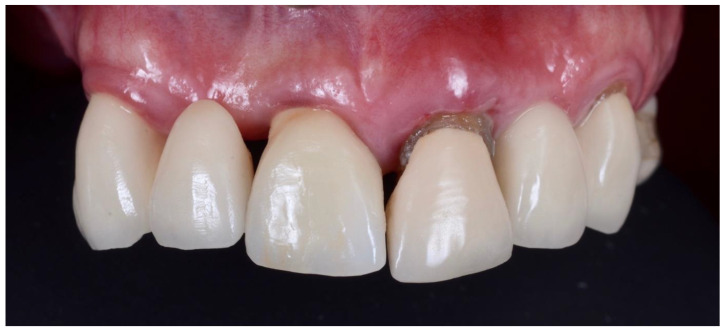
Preoperative photograph of PFM post-core crown (21), with associated buccal root fracture.

**Figure 2:**
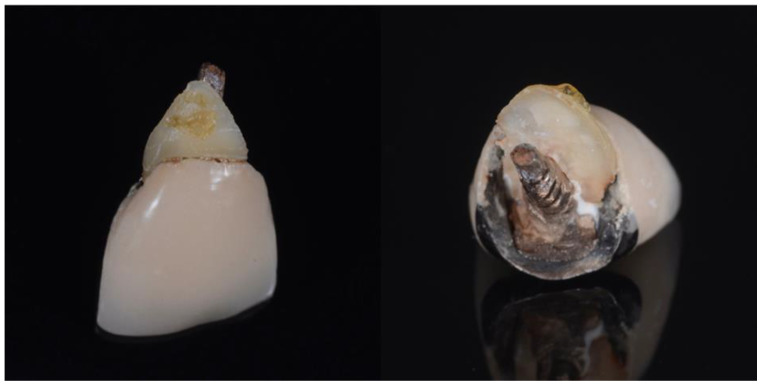
Photographs of post-core crown (21) with fractured piece of buccal root still firmly attached.

**Figure 3:**
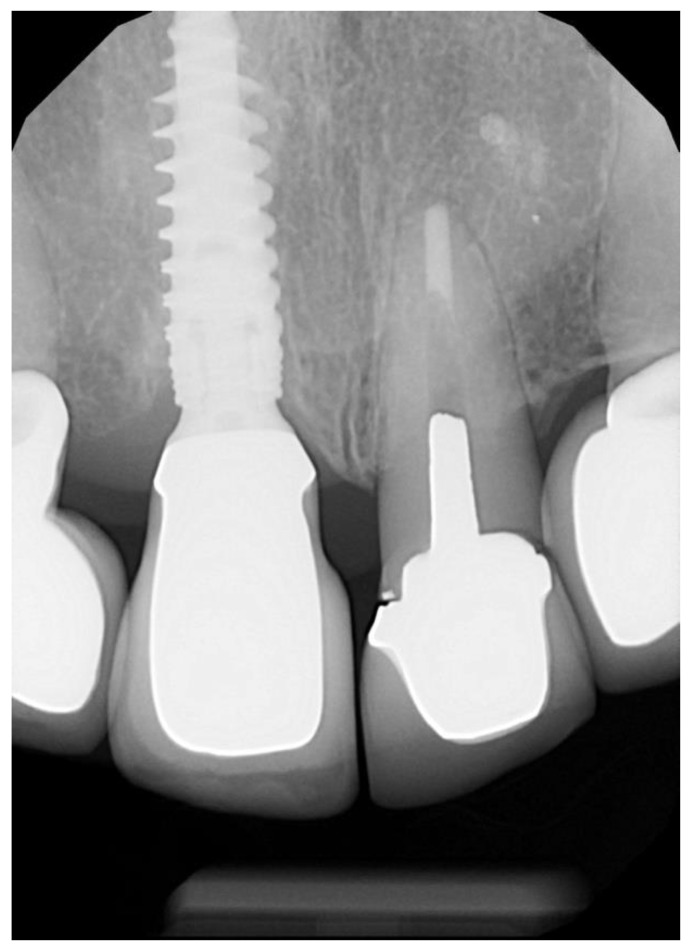
Pre-operative X-ray of 21 prior to shield preparation.

**Figure 4:**
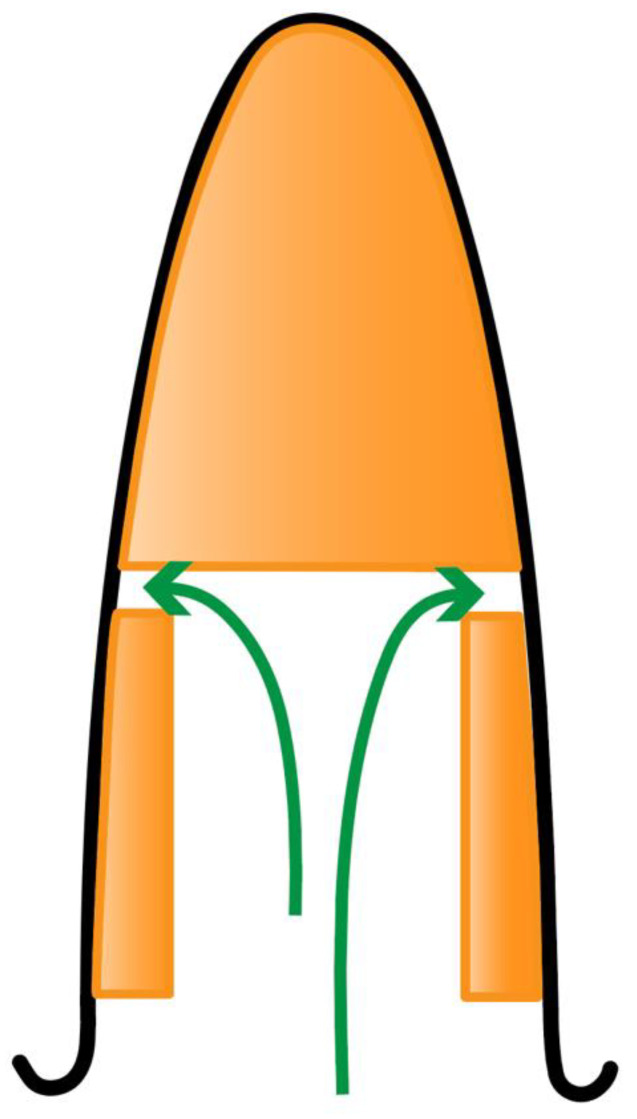
Apex is completely amputated from coronal preparation.

**Figure 5:**
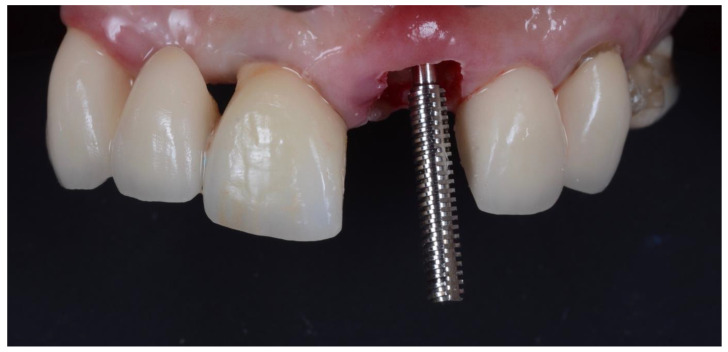
Insertion of root screw into apex of sectioned root.

**Figure 6:**
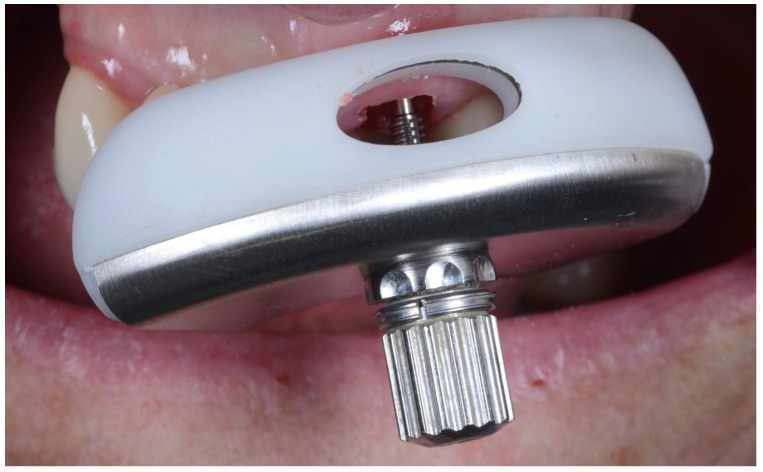
Loosening of root apex using the root extractor.

**Figure 7:**
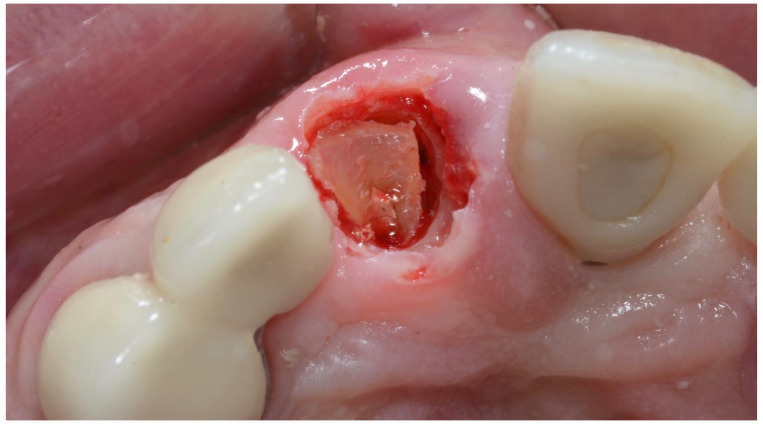
Removal of root apex through the lumen of the 360-degree socket-shield.

All surgeries were performed by the author. The surgical area was anesthetised with Articaine (Septanest 1:100,000, Septodont, Saint-Maur-des-Fossés, France) following photographic and radiographic documentation (Figure 1, Figure 2 and Figure 3). The tooth was decoronated and then hollowed out coronally by following the root outline. The apical portion of the root was then amputated and completely removed (Figure 4, Figure 5, Figure 6 and Figure 7). For multi-rooted teeth the same process was repeated for each root. In the case of acute or chronic apical infection, the socket was debrided with a curette and copious saline rinses used. All procedures were performed flapless. 

**Figure 8:**
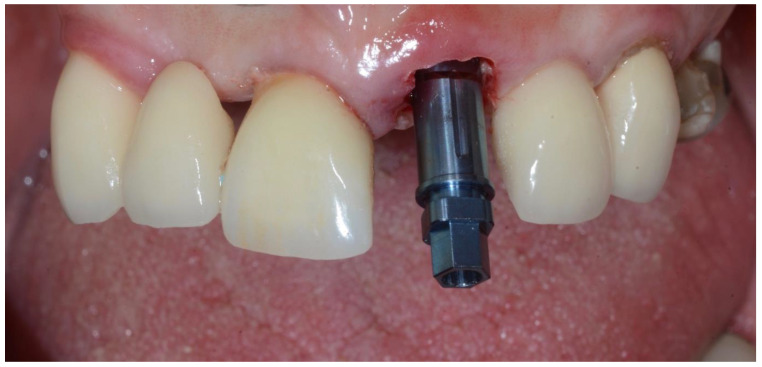
Insertion of implant to a depth of 3–4 mm below the mid-buccal gingival margin.

**Figure 9:**
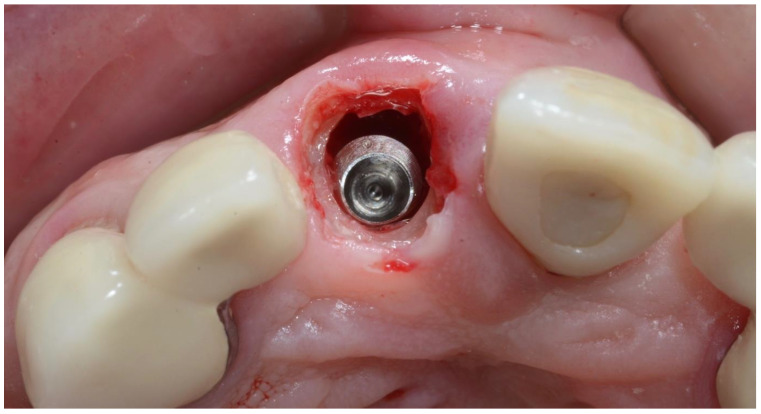
Occlusal view of implant showing intimate relationship with the 360-degree socket-shield.

The resultant 360-degree shield was approximately 1 ± 0.5 mm in thickness. The osteotomy was then prepared free-handed without the use of any guides. The implant was then inserted into the alveolar bone through the 360-degree shield at a depth of 3–4 mm below the mid-buccal gingival margin (Figure 8 and Figure 9). 

In cases of vertical root fractures, the crack line was debrided with a long diamond fissure bur to remove any biofilms. Where a section of shield was mobile, xenograft particles (Bio-Oss, Geistlich, Wolhusen, Switzerland) were packed tightly between the implant and the shield for stability. Buccal fenestrations were treated internally with a membrane (Bio-Gide, Geistlich), and then Bio-Oss packed in against it. For patients with active periodontal disease, the shield was prepared below the level of visible contamination of the root surface at bone level.

**Figure 10:**
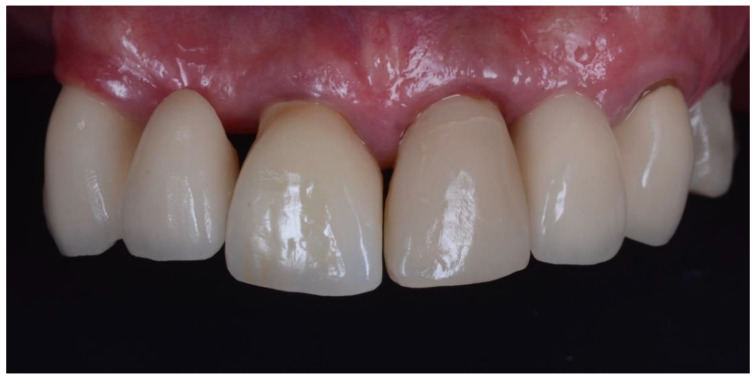
Temporary crown at one week review.

At the time of surgery, a temporary abutment was fitted to the implant. Flowable resin (Filtek Supreme XTE, 3M ESPE, Maplewood, MN, USA) was then adapted around the temporary cylinder and used to cover the shield. For posterior teeth (25 in total) the height of the cylinder was kept close to gingival level. For anterior teeth (6 in total), the temporary cylinder was left longer and a provisional crown (Integrity, Dentsply, York, PA, USA) constructed using a putty key (Elite HD+, Zhermack, Badia Polesine, Italy). Provisional crowns were reduced slightly into infra-occlusion to avoid any centric and eccentric contacts (Figure 10).

At the conclusion of the surgical appointment, patients were given 400 mg Ibuprofen (Apo-Ibuprofen 400, Apotex, Toronto, ON, Canada) and 2g Amoxycillin (Apo-Amoxycillin, Apotex, Toronto, ON, Canada). Patients were then given post-operative instructions and advised to avoid chewing on the affected area for 6 weeks. They were then prescribed a full course of Amoxycillin and advised to take Ibuprofen every 6–8 h as necessary. Patients were reviewed immediately one week after surgery.

**Figure 11:**
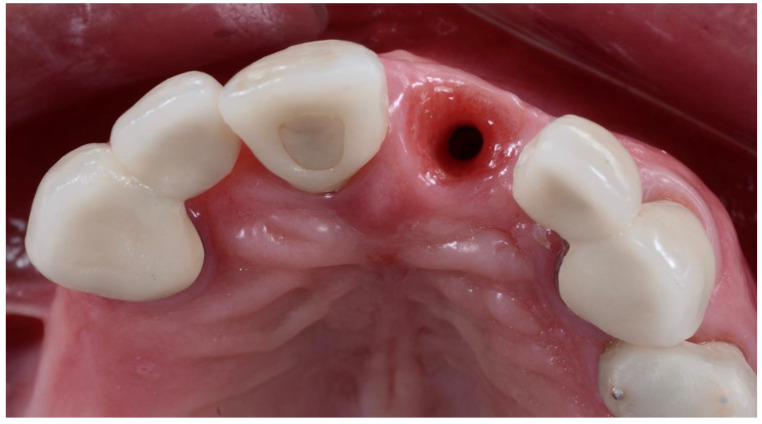
Occlusal view at crown impression appointment after temporary crown removal.

**Figure 12:**
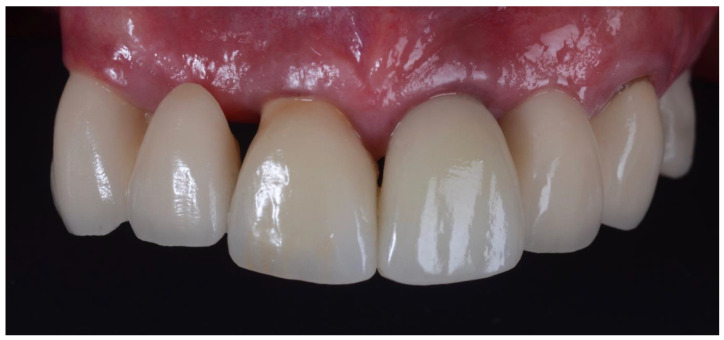
Final crown of 21 at one week after insertion. This case is symmetrical with cantilever bridges on both canines supporting a lateral pontic. 11 is also an implant crown that was placed using a traditional technique, with bovine bone in the jumping gap. Following 11’s replacement, significant recession occurred on the adjacent pontic 12. Prior to the complete extraction of 11, the pontic on 12 was gingivally-fitted similar to 22.

The temporary prosthetic was removed (Figure 11) approximately 6–12 weeks after implant placement and crown impressions were taken. All implants in the study integrated uneventfully, and successful integration was confirmed with a resonance frequency analysis (Osstell, W&H, Bürmoos, Austria). Three anterior crowns were cement-retained and the other 28 cases were screw-retained. Anterior crowns were made of lithium disilicate with custom zirconia over a titanium base. Posterior teeth were made from monolithic zirconia over a titanium base. A review appointment occurred one week after the crown insert (Figure 12). 

### 2.2. Complications

There were two cases of shield exposure both involving posterior teeth when the provisional was removed. The shield was reduced with a round diamond bur and a healing abutment was then inserted into the implant to encourage gingival coverage of the exposed shield. Two weeks later, the patient was recalled and the final crown was inserted uneventfully.

### 2.3. Data Accumulation

**Figure 13:**
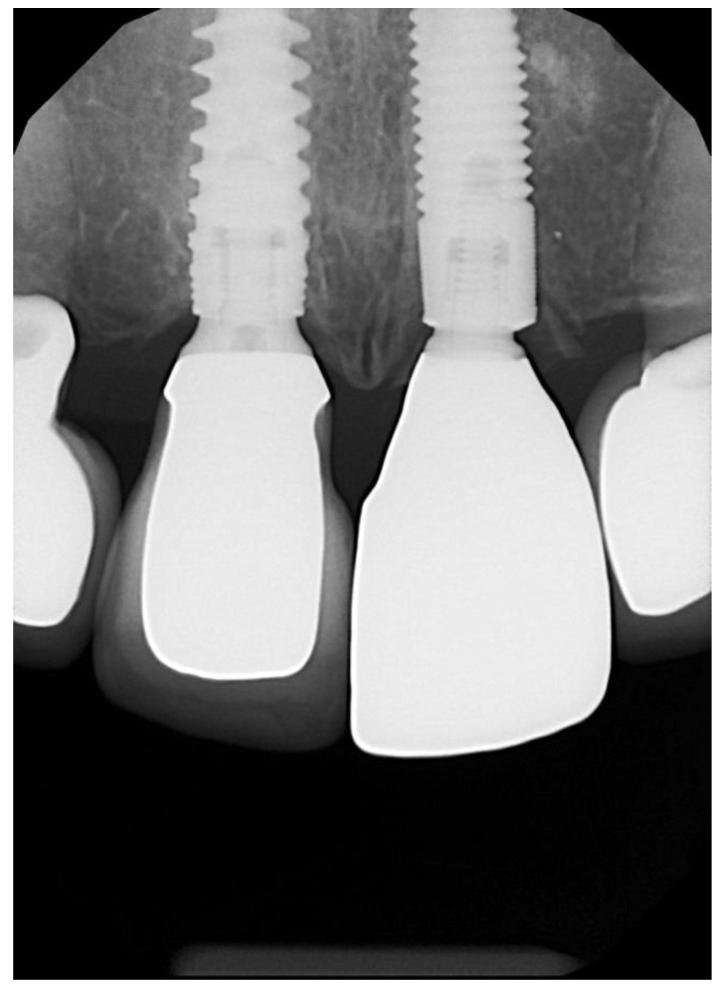
X-ray of final crown confirming correct seating and no interference with the shield.

**Figure 14:**
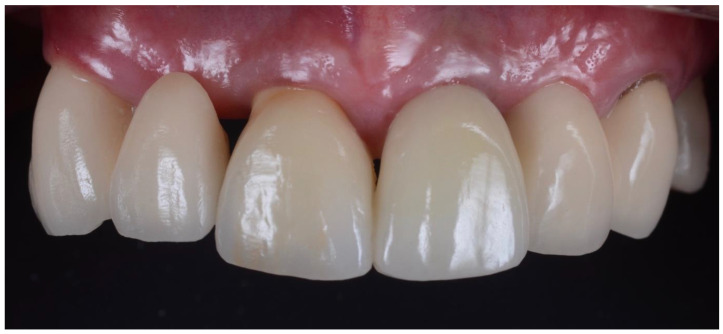
Subsequent review appointment showing complete resolution of inflammation around the crown (21). Minimal recession around pontic (22) also noted.

A final review appointment was organised for all patients that were included in this study and post-operative silicone impressions (Imprint 4, 3M ESPE), clinical photos and radiographs were taken (Figure 13 and Figure 14). A staff member provided each patient with a questionnaire. This questionnaire incorporated a Visual Analogue Scale (VAS) relating to pain, bleeding, swelling and satisfaction. The duration of both pain and swelling post-operatively was also recorded on this form.

All impressions taken before treatment and at the final review were poured in a type IV die stone (Fujirock EP, GC, Tokyo, Japan). Models were than scanned with an optical scanner (Emerald 2, Planmeca, Helsinki, Finland). The Standard Tessellation Language (STL) files were then imported into a digital imaging software program (SMOP Volume Compare, Swissmeda, Baar, Switzerland) and the data sets from the pre-extraction and post-treatment merged. Volumetric analysis was conducted on both the buccal and lingual surfaces. Measurements were made parallel to the tooth axis using the pre-extraction gingival zenith as a reference point. These changes were recorded as the mean loss in distance (Δd [mm] = Δvol [mm^3^]/area [mm^2^]) in accordance with previous studies [22,23]. Cases were rejected if the models were incomplete or unclear. Volumetric measurements were performed by both SB and HA working together. 

Recession of the soft tissues was calculated by measuring the vertical distance change at both the mid-buccal and mid-lingual gingival margin of the superimposed models.

Papillary height changes were determined using available photographs taken prior to treatment and at the final review. Measurements were made parallel to the long axis of the tooth using a digital caliper. Reference points from the stone models were then used to calibrate these measurements. Where the papilla was not clearly visible on the photograph, or the angles in which the photographs were taken were significantly different, the cases were excluded from papillary height measurements. All papillary height measurements were performed by one operator (CC). 

### 2.4. Statistical Analysis

A null hypothesis was tested for each of the following six outcome variables: Mean Distance (MD) Buccal, Mean Distance (MD) Lingual, Mid-Buccal Recession, Mid-Lingual Recession, Mesial Papillary Recession and Distal Papillary Recession. The null hypothesis tested was the following: There is no volumetric difference between the baseline and the volumetric review.

This null hypothesis was rejected if α < 0.05 in the Wilcoxon sign test (a non-parametric approach), comparing the cohort median with a hypothesised median of 0. 

The analysis was repeated for tooth locations within each outcome variable.

Statistical analysis was performed using STATA V16 (Texas 77845-4512, USA, Dallas TX, USA).

Descriptive statistics were used to analyse patient-reported outcomes. 

## 3. Results

**Figure 15:**
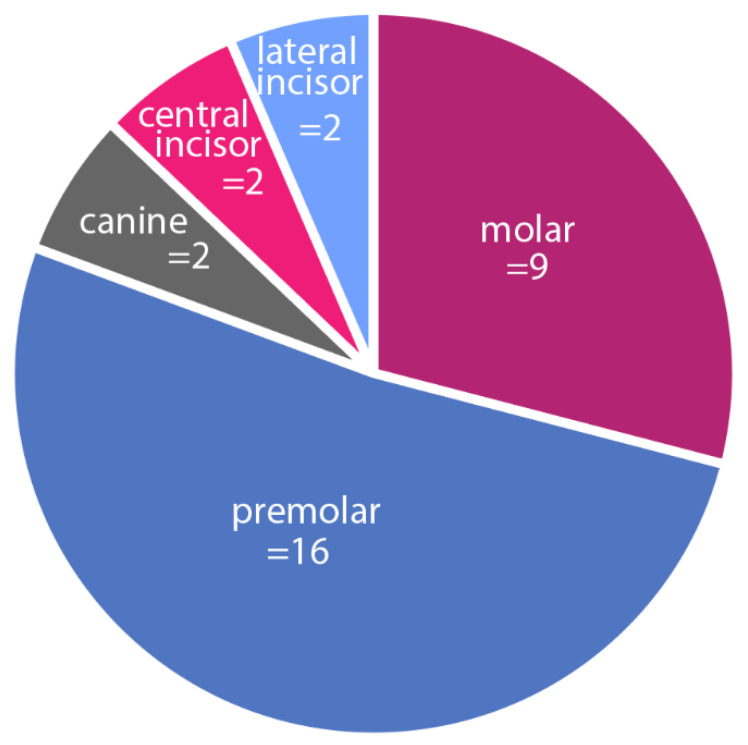
Distribution of immediate implant sites.

Distribution of the sites is shown in Figure 15. Mean loss of buccal and lingual tissue was −0.30 ± 0.32 mm and −0.17 ± 0.27 mm, respectively (Table 2). The mean loss of buccal tissue is statistically significant at the α = 0.05 level (*p* < 0.001). The mean loss of lingual tissue is not statistically significant at the α = 0.05 level (*p* = 0.664). 

Mean recession at the mid-buccal and mid-lingual gingival margins was −0.66 ± 0.64 mm and −0.87 ± 0.84 mm, respectively. Mean recession of the mesial and distal papilla was −0.26 ± 0.55 mm and −0.29 ± 0.52 mm, respectively (Table 3). A summary of the results for the different tooth sites is detailed in Table 4. 

Patients reported a high degree of satisfaction of 97.74 ± 5.60% with this procedure. Patients did not report any post-operative bleeding and took ibuprofen for a mean average of 0.55 ± 0.68 days following the procedure. Post-operative swelling was rated at 98.71 ± 4.99% satisfaction using a Visual Analogue Scale (VAS), with a mean duration of 0.13 ± 0.5 days. Post-operative pain was rated at 91.13 ± 8.14% satisfaction, using a VAS, with a mean duration of 0.82 ± 0.82 days (Table 5). 

## 4. Discussion

Volumetric analysis showed minimal changes to the buccal tissues with a mean loss of −0.30 mm. There are very few volumetric studies reported in the literature using conventional implant techniques. Such comparisons between socket-shield and other techniques with the limited available data are summarised in Table 6. 

In the current study the mean distance change on the lingual was minimal at only 0.17 mm. Aslan [15] reported significant palatal atrophy of −1.21 mm at 1 mm below the gingival margin using only a buccal shield. The volumetric stability in the palatal region in the current study using a 360-degree shield indicates a biological advantage over a partial shield, but more studies need to be conducted.

Mid-buccal gingival recession calculated at −0.66 mm was higher than the −0.33 mm reported by Bäumer et al. [5]. There are a few explanations for this. Firstly, the depth of the shield relative to the bone significantly influences the final gingival position [3]. As the study by Bäumer et al. and the current study were performed flapless, it is difficult to ascertain the exact position of shield relative to the bone [5]. Tolerance may be up to 1 mm. Carnevale et al. found that preparing the shield to bone level would result in a 1 mm resorption of buccal bone [24]. 

**Table 6:** Immediate implants comparing socket-shield techniques to traditional techniques.

Technique		Author	Length of Study (Years)	Mesial Papillary Recession (mm)	Distal Papillary Recession (mm)	MD Buccal Recession	Mid-Buccal Recession (mm)	MD Buccal Bone Loss (mm)
**Socket-Shield**	360-degree socket shield	Cameron Castle 2022	2	−0.26 ± 0.55	−0.29 ± 0.52	−0.30 ± 0.32	−0.66 ± 0.64	n.a.
	Buccal socket shield	Bäumer et al., 2017 [5]	5	n.a.	n.a.	−0.37 ± 0.30	−0.33 ± 0.23	n.a.
**Traditional**	No graft	Gavilán R 2017 [25]	1	−0.89 ± 0.41	−0.84 ± 0.50	−0.71 ± 0.35	−1.10 ± 0.64	n.a.
	Bovine bone in gap	Gavilán R 2017 [25]	1	−0.95 ± 0.62	−0.84 ± 0.46	−0.79 ± 0.44	−0.82 ± 0.53	n.a.
	Bovine bone in gap	Van Nimwegen et al., 2018 [26]	1	n.a.	n.a.	−0.49 ± 0.54	−0.48 ± 1.13	−0.47 ± 0.55
	Bovine in gap + CTG on buccal	Zuiderveld et al., 2020 [27]	1	n.a.	n.a.	−0.68 ± 0.59	0.20 ± 0.70	−0.81 ± 0.66

MD = Mean Distance; n.a. = data not reported on.

**Figure 16:**
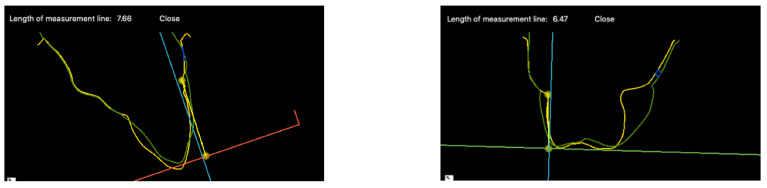
Superimposed digital scans showing a larger emergence profile of the final implant crown (green) than the original tooth (yellow).

**Figure 17:**
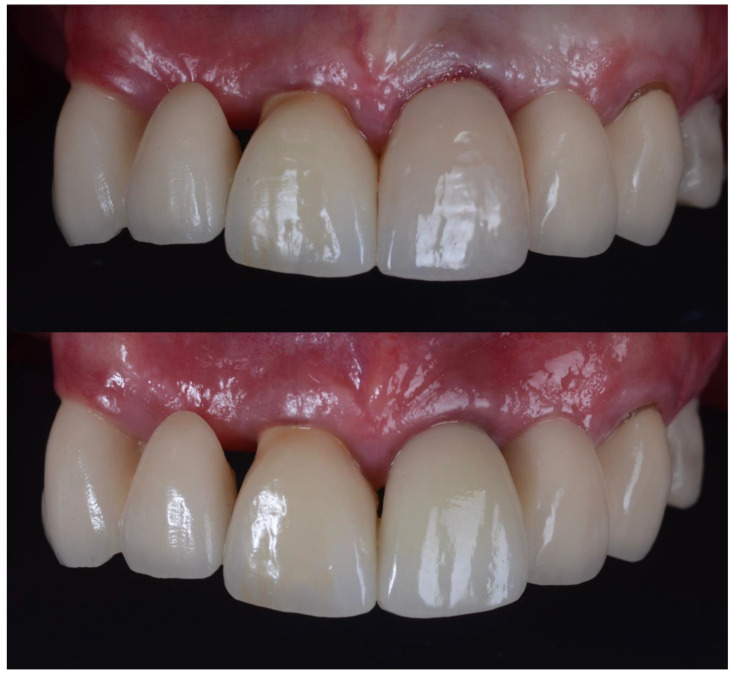
(**Top**) Photograph shows crown before colour and emergence profile have been adjusted. (**Bottom**) Photograph shows less gingival recession when emergence profile of crown has been significantly reduced.

The other variable influencing the buccal gingival margin is the prosthetics. Over contoured crowns at the gingival level may negatively affect the relative gingival height (Figure 16). A thickened emergence profile can effectively displace the gingival margin in an apical direction creating a recession (Figure 17). 

**Figure 18:**
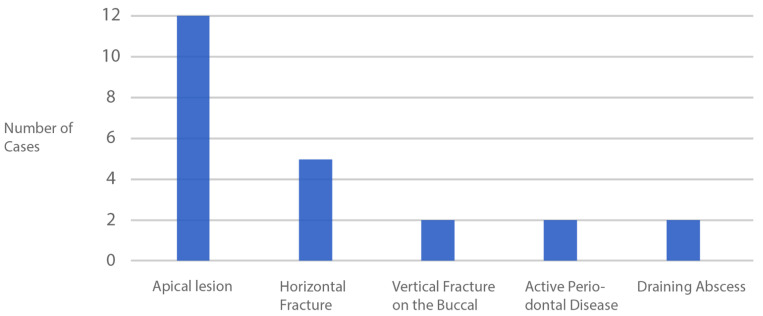
Incidence of more severe conditions affecting some of the cases in this study.

In this current study, 39% of the cases had apical lesions but there were no post-operative complications relating to unresolved infection (Figure 18). All intraoral radiographs taken at the final review demonstrated a complete resolution of the lesion. This newer technique may prove to be more predictable at completely removing the apex versus current socket-shield techniques. In a study by Siormpas et al., the apex of the shield was not completely removed in one patient and resorption occurred over time [28].

Two of the 31 cases were performed on periodontally affected tooth roots. The shields were reduced to bone level where they should be below the level of biofilm accumulation. In theory, if the retained shields remain submerged, they should not be exposed to bacterial contamination that can trigger periodontal disease [29]. Longer term follow-up is required to ascertain if these cases remain stable and infection-free over time.

The question remains if reducing a shield to bone level, as suggested by Gluckman et al., will safeguard against future migration [20]. According to Zuhr et al., this issue of shield migration is more complicated, and he has postulated that the continual anterior-caudal growth of the maxilla may be the underlying cause [21,30]. There may be other reasons to explain this process of shield migration. It could just be a genetic phenomenon affecting certain individuals, whereby an unrestrained shield naturally migrates to the surface of the gingiva. One such complication of shield migration and subsequent removal was documented by Zuhr et al. [21] He suggested “locking” the implant to the shield. A 360-degree socket-shield provides more available surface area to achieve this. This is an important advantage.

Anterior teeth and premolars are much easier to “lock” the implant to the coronal aspect of the shield using the CWST. Molars are more difficult as the implant will not normally contact the shield unless the implant is positioned asymmetrically, or a wider diameter implant is selected. 

According to the study by Bäumer et al., a vertical root fracture on the buccal aspect was a contraindication for a socket-shield [5]. Buccal vertical root fractures were also a contraindication in other studies [20,22,31]. This may be related to the prepared shield being inherently unstable with a crack through it, regardless of whether disinfection was achievable. Such root fractures were not a contraindication in the current study. By preparing a 360-degree socket-shield in root fracture cases, the possibility that more rigid and viable sections of shield are retained is increased. This is a clear advantage should part of a shield be lost to migration over time.

This technique should be reserved for experienced operators that are familiar with immediate implant procedures. With practice, the CWST for preparing a 360-degree socket-shield on an anterior tooth can become both efficient and predictable. The need for expensive and painful grafting procedures with compromised aesthetics can be avoided. Excellent illumination and magnification are essential. Large single-rooted anterior teeth are much simpler to prepare than multi-rooted teeth.

The reported findings are based on data from a volumetric study. Data accumulated from volumetric analyses while precise can be subject to various procedural inaccuracies as reported by Hinze et al. [22]. 

### Future Studies

The fact that bone levels were not reported on was a limiting factor in the current study. This was because the available radiographs taken before and after treatment were not directly comparable due to angulation discrepancies. The intraoral periapical radiographs did however demonstrate bone formation around all implants and successful integration was further confirmed using resonance frequency analysis. Future CBCT studies would be beneficial. 

## 5. Conclusions

The results of this study showed excellent soft tissue stability and aesthetics were achieved using the CWST, with minimal postoperative pain. The other main advantage of retaining a 360-degree socket-shield, is there is more available surface area to lock the implant to the shield to prevent shield migration over time. 

## Figures and Tables

**Table 1:** Patient selection criteria.

Inclusion Criteria
Medically healthy adult
Vertical fracture including on the buccal aspect
Horizontal fractures at or below bone level
Acute and chronic apical infection
Acute periodontal disease
Informed consent
Immediate implant using CastleWall Surgical Technique
**Exclusion Criteria**
Primary stability <25 Ncm
Patient not available for final review
Models not taken prior to treatment
Models not clear or incomplete around treatment site

**Table 2:** Volumetric analyses measured as the mean distance (MD) change in both the buccal and lingual directions. Recession at both the mid-buccal and mid-lingual gingival margins was also reported.

Case No	Site (FDI)	MD Buccal (mm)	MD Lingual (mm)	Mid-Buccal Recession (mm)	Mid-Lingual Recession (mm)
**1**	17	−0.77	−0.69	−1.40	−1.00
**2**	22	0	n.a	−0.76	n.a.
**3**	24	−0.22	0.21	0.32	−0.34
**4**	22	−0.43	0.02	−1.27	−0.89
**5**	24	−0.29	−0.37	−0.36	−0.48
**6**	16	0.08	n.a.	−0.28	n.a.
**7**	14	−0.10	0.03	−1.61	−0.25
**8**	35	0.18	−0.26	−0.51	−0.52
**9**	11	−0.24	0.02	0.31	0.57
**10**	33	−0.18	n.a.	−0.92	−0.02
**11**	26	n.a.	n.a.	n.a.	n.a.
**12**	44	−0.15	0.04	−0.87	−0.70
**13**	26	−0.78	n.a.	−0.94	−1.22
**14**	25	0.10	−0.21	−1.71	−1.48
**15**	24	0.20	−0.03	−1.48	−0.66
**16**	26	−0.98	n.a.	−0.93	−2.99
**17**	26	−0.20	−0.68	0.58	−1.75
**18**	23	−0.09	−0.18	−0.77	−0.48
**19**	26	−0.62	n.a.	−1.57	−2.02
**20**	24	−0.32	−0.07	0.24	−0.85
**21**	21	−0.87	−0.30	−1.13	−2.43
**22**	25	−0.44	0.01	−1.29	−0.54
**23**	35	−0.28	n.a.	−0.51	−0.74
**24**	16	−0.32	0.04	−0.35	−0.02
**25**	36	−0.12	−0.56	0.26	−0.93
**26**	44	−0.51	n.a.	−0.18	n.a.
**27**	35	−0.61	−0.64	−0.90	−1.69
**28**	14	−0.87	0.07	−1.01	−1.72
**29**	14	−0.13	0.06	−0.80	0.39
**30**	45	0.02	−0.06	0.07	0
**31**	25	−0.15	n.a.	−0.14	−0.66

n.a. = not applicable as data not available.

**Table 3:** Changes to the mesial and distal papilla height.

Case No	Site (FDI)	Pre-op Mesial (mm)	Post-op Mesial (mm)	Mesial Recession (mm)	Pre-op Distal (mm)	Post-op Distal (mm)	Distal Recession (mm)
**1**	17	n.a.	n.a.		n.a.	n.a.	
**2**	22	4.25	4.22	−0.03	2.99	2.92	−0.24
**3**	24	3.92	3.91	−0.01	3.43	2.92	−0.51
**4**	22	5.85	4.50	−1.35	4.34	3.15	−1.19
**5**	24	4.92	5.03	0.11	3.41	3.31	−0.10
**6**	16	2.60	2.22	−0.38	3.17	2.46	−0.71
**7**	14	3.13	3.42	0.29	1.35	1.91	0.56
**8**	35	2.94	2.93	−0.01	2.84	2.97	0.13
**9**	11	4.27	3.74	−0.53	3.46	2.38	−1.08
**10**	33	2.81	2.52	−0.29	1.29	1.64	0.35
**11**	26	n.a.	n.a.		n.a.	n.a.	
**12**	44	n.a.	n.a.		n.a.	n.a.	
**13**	26	n.a.	n.a.		n.a.	n.a.	
**14**	25	n.a.	n.a.		n.a.	n.a.	
**15**	24	n.a.	n.a.		n.a.	n.a.	
**16**	26	3.00	2.80	−0.20	2.09	2.43	0.34
**17**	26	n.a.	n.a.		n.a.	n.a.	
**18**	23	3.26	2.97	−0.29	2.36	2.29	−0.07
**19**	26	n.a.	n.a.		n.a.	n.a.	
**20**	24	4.87	4.07	−0.80	2.62	2.65	0.03
**21**	21	5.96	6.56	0.60	5.48	5.29	−0.19
**22**	25	n.a.	n.a.		n.a.	n.a.	
**23**	35	n.a.	n.a.		n.a.	n.a.	
**24**	16	n.a.	n.a.		n.a.	n.a.	
**25**	36	n.a.	n.a.		n.a.	n.a.	
**26**	44	n.a.	n.a.		n.a.	n.a.	
**27**	35	n.a.	n.a.		n.a.	n.a.	
**28**	14	5.53	4.01	−1.52	4.09	3.04	−1.05
**29**	14	4.73	4.88	0.15	3.09	2.80	−0.29
**30**	45	4.22	3.98	−0.24	3.64	3.35	−0.29
**31**	25	0.99	1.03	0.04	1.86	1.22	−0.64

n.a. = not applicable as data not available.

**Table 4:** Summary of volumetric gingival margin and papillary changes from pre-treatment to final review.

Variable	*n*	Mean ± SD	Median (IQR)	*p*-Value *
**MD Buccal**	**30**	**−0.30 ± 0.32**	**−0.23 (0.41)**	**<0.001**
Molar	8	−0.46 ± 0.38	−0.47 (0.62)	
Premolar	16	−0.22 ± 0.29	−0.19 (0.34)	
Incisor	6	−0.30 ± 0.31	−0.21 (0.34)	
**MD Lingual**	**21**	**−0.17 ± 0.27**	**−0.06 (0.33)**	**0.664**
Molar	4	−0.47 ± 0.35	−0.62 (0.43)	
Premolar	13	−0.09 ± 0.23	−0.03 (0.25)	
Incisor	4	−0.11 ± 0.16	−0.80 (0.26)	
**Mid-buccal Recession**	**30**	**−0.66 ± 0.64**	**−0.79 (0.95)**	**0.001**
Molar	8	−0.58 ± 0.77	−0.64 (1.16)	
Premolar	16	−0.67 ± 0.65	−0.66 (0.99)	
Incisor	6	−0.76 ± 0.56	−0.85 (0.37)	
**Mid-lingual Recession**	**27**	**−0.87 ± 0.84**	**−0.70 (1.14)**	**<0.001**
Molar	7	−1.42 ± 0.94	−1.22 (1.09)	
Premolar	15	−0.68 ± 0.58	−0.66 (0.51)	
Incisor	5	−0.65 ± 1.13	−0.48 (0.87)	
**Mesial Papillary Recession**	**17**	**−0.26 ± 0.55**	**−0.20 (0.42)**	**0.144**
Molar	2	−0.29 ± 0.13	−0.29 (0.18)	
Premolar	9	−0.22 ± 0.58	−0.01 (0.35)	
Incisor	6	−0.32 ± 0.64	−0.30 (0.31)	
**Distal Papillary Recession**	**17**	**−0.29 ± 0.52**	**−0.24 (0.67)**	**0.144**
Molar	2	−0.19 ± 0.74	−0.19 (1.05)	
Premolar	9	−0.24 ± 0.47	−0.29 (0.54)	
Incisor	6	−0.40 ± 0.60	−0.22 (1.01)	

MD = Mean Distance; SD = Standard deviation; IQR = inter quartile range; * = Wilcoxon sign test.

**Table 5:** Patient-reported outcomes using a questionnaire which included a Visual Analogue Scale (VAS).

Case No.	Tooth No	Bleeding * (%)	Swelling * (%)	Pain * (%)	Satisfaction (%)	Swelling (Days)	Pain (Days)	Analgesics (Days)
**1**	17	100	100	90	100	0	1	0
**2**	22	100	100	100	100	0	0	0
**3**	24	100	80	90	100	2	1	1
**4**	22	100	100	80	100	0	2	1
**5**	24	100	100	80	100	0	2	1
**6**	16	100	100	90	100	0	1	1
**7**	14	100	100	100	100	0	0	0
**8**	35	100	100	90	100	0	2	0
**9**	11	100	100	100	100	0	0	2
**10**	33	100	100	80	90	0	2	0
**11**	26	100	100	80	90	0	2	0
**12**	44	100	100	90	90	0	1	1
**13**	26	100	100	90	100	0	0	0
**14**	25	100	100	80	80	0	2	1
**15**	24	100	100	80	80	0	2	1
**16**	26	100	100	100	100	0	0	0
**17**	26	100	100	90	100	0	1	1
**18**	23	100	100	100	100	0	0	0
**19**	26	100	100	100	100	0	0	0
**20**	24	100	80	90	100	2	1	2
**21**	21	100	100	90	100	0	0.5	0
**22**	25	100	100	80	100	0	1	1
**23**	35	100	100	100	100	0	0	0
**24**	16	100	100	90	100	0	2	2
**25**	36	100	100	100	100	0	0	0
**26**	44	100	100	100	100	0	0	0
**27**	25	100	100	80	100	0	1	1
**28**	14	100	100	100	100	0	0	0
**29**	14	100	100	100	100	0	0	0
**30**	45	100	100	85	100	0	1	1
**31**	25	100	100	100	100	0	0	0
**Mean**		100	98.71	91.13	97.74	0.13	0.82	0.55
**Median**		100	100	90	100	0	1	0
**SD**		0	4.99	8.14	5.60	0.50	0.82	0.68

SD = Standard Deviation; * = results from Visual Analogue Scale (VAS). Total dissatisfaction expressed as 0% and complete satisfaction expressed as 100%.
